# Haemogram-Derived Indices for Screening and Prognostication in Critically Ill Septic Shock Patients: A Case-Control Study

**DOI:** 10.3390/diagnostics10090638

**Published:** 2020-08-27

**Authors:** Piotr S. Liberski, Michał Szewczyk, Łukasz J. Krzych

**Affiliations:** Department of Anaesthesiology and Intensive Care, Faculty of Medicine in Katowice, Medical University of Silesia, 40752 Katowice, Poland; pliberski@sum.edu.pl (P.S.L.); michal.szewlek@gmail.com (M.S.)

**Keywords:** sepsis, critical illness, biomarker, screening, prognostication

## Abstract

This study aimed (1) to assess the diagnostic accuracy of neutrophil-to-lymphocyte (NLR), platelet-to-lymphocyte (PLR), monocyte-to-lymphocyte (MLR) and platelet count-to-mean platelet volume (PLT/MPV) ratios in predicting septic shock in patients on admission to the intensive care unit (ICU) and (2) to compare it with the role of C-reactive protein (CRP), procalcitonin (PCT) and lactate level. We also sought (3) to verify whether the indices could be useful in ICU mortality prediction and (4) to compare them with Acute Physiology and Chronic Health Evaluation II (APACHE II), Simplified Acute Physiology Score II (SAPS II) and Sequential Organ Failure Assessment (SOFA) scores. This retrospective study covered 138 patients, including 61 subjects with multi-organ failure due to septic shock (study group) and 77 sex- and age-matched controls. Septic patients had significantly higher NLR (*p* < 0.01) and NLR predicted septic shock occurrence (area under the ROC curve, AUROC = 0.66; 95% CI 0.58–0.74). PLR, MLR and PLT/MPV were impractical in sepsis prediction. Combination of CRP with NLR improved septic shock prediction (AUROC = 0.88; 95% CI 0.81–0.93). All indices failed to predict ICU mortality. APACHE II and SAPS II predicted mortality with AUROC = 0.68; 95% CI 0.54–0.78 and AUROC = 0.7; 95% CI 0.57–0.81, respectively. High NLR may be useful to identify patients with multi-organ failure due to septic shock but should be interpreted along with CRP or PCT. The investigated indices are not related with mortality in this specific clinical setting.

## 1. Introduction

Sepsis is an abrupt life-threatening organ dysfunction with dangerously high mortality, when accompanied by shock and multi-organ failure [1]. Although the pathophysiology of sepsis remains complex and not fully elucidated, dysregulated host response to infection lies at its basis. Proper early diagnosis improves prognosis by increasing the probability of implementing adequate treatment, which predominantly consists of prompt antibiotic therapy and elimination (or control) of the source of infection [2]. Looking for relevant tools to identify patients with sepsis and septic shock, scientific research focuses on evaluating the role of demographic, clinical and laboratory markers. So far, several diagnostic tools have been suggested in screening [3,4,5].

Initially, much attention was paid to the analysis of the parameters of the systemic inflammatory response syndrome (SIRS) but it is now known that the occurrence of SIRS does not confirm the presence of sepsis, thus this criterion has been removed from international recommendations [1]. Instead, investigations have been directed into the occurrence of multi-organ failure or its key objective surrogates: hypotension, tachypnoe or altered consciousness, which are components of a simple screening scale called ‘Quick Sequential Organ Failure Assessment (qSOFA)’ [1]. Suspicion of sepsis should be cautiously verified in the course of further diagnostics and effective treatment should be applied as soon as possible. To facilitate decision-making in sepsis and septic shock, suitable guidelines have been developed, which are regularly updated [2].

In recent years, several haemogram-derived indices have been suggested for screening and prognostication in bacteraemia and sepsis [6,7,8,9]. To improve screening, it has been suggested to assess them concomitantly with some well-known markers of systemic inflammation, including leucocytosis, C-Reactive Protein (CRP) or procalcitonin (PCT) [10,11]. To improve prognostication, they could be used in addition to some well-known ICU scorings, including APACHE II (Acute Physiology and Chronic Health Evaluation II), SAPS II (Simplified Acute Physiology Score II) and SOFA (Sequential Organ Failure Assessment) [12,13].

In this study, we aimed (1) to assess the diagnostic accuracy of neutrophil-to-lymphocyte (NLR), platelet-to-lymphocyte (PLR), monocyte-to-lymphocyte (MLR) and platelet count-to-mean platelet volume (PLT/MPV) ratios in predicting septic shock in critically ill patients on admission to the intensive care unit (ICU) and (2) to compare them with the role of CRP, PCT and lactate level. We also sought (3) to verify the diagnostic accuracy of NLR, PLR, MLR and PLT/MPV in ICU mortality prediction in patients with septic shock and (4) to compare them with the role of APACHE II, SAPS II and SOFA scores.

## 2. Material and Methods

This retrospective case-control study covered 138 patients who were hospitalized in a mixed 10-bed ICU in 2019. The study group comprised 61 subjects who developed multi-organ failure due to septic shock. For diagnosis of sepsis and septic shock, we applied the third international definition and appropriate diagnostic criteria [1]. Multi-organ failure was defined as the development of potentially reversible physiologic derangement involving two or more organ systems and arising in the wake of a potentially life-threatening physiologic insult. The control group comprised 77 sex- and age-matched patients who were admitted to the ICU due to non-infectious reasons (traumatic or non-traumatic brain injury: *n* = 34; acute respiratory failure: *n* = 17; acute cardiac failure: *n* = 11; metabolic disequilibrium, including acute kidney injury: *n* = 3, intoxication: *n* = 3, acute liver failure: *n* = 1; haemorrhagic shock: *n* = 8). Neither sample size calculation nor power analysis was performed a priori.

Demographic, clinical and laboratory data were retrieved from medical records. APACHE II, SAPS II and SOFA scores were calculated based on the worst values within 24 h post admission. Lactate levels (mmol/L) were measured in arterial blood samples in the ICU (RAPIDPoint^®^ 500, Erlangen, Germany). CRP (mg/L) (Olympus AU680^®^, Bellport, NY, USA), PCT (ng/mL) (Roche Cobas601^®^, Basel, Switzerland) and haemogram-derived indices (Sysmex^®^ XN1000, Norderstedt, Germany) were assessed in the central laboratory in venous blood samples retrieved shortly post-ICU admission. NLR, PLR, MLR, and PLT/MPV were subsequently calculated. ICU mortality was considered as the outcome. Data were always collected and verified independently by two researchers.

Due to the retrospective, non-interventional and anonymous nature of the survey, no approval of the Ethics Committee was required. The STROBE Statement (Strengthening The Reporting of OBservational studies in Epidemiology) was applied for appropriate reporting.

Statistical analysis was performed using MedCalc Statistical Software version 17.2 (MedCalc Software bvba, Ostend, Belgium). Continuous variables were expressed as median and interquartile range (IQR). Qualitative variables were expressed as absolute values and/or percent. Between-group differences for quantitative variables were assessed using the Mann–Whitney *U*-test, after verification of variables’ distribution with the Shapiro–Wilk test. The chi-squared or Fisher′s exact test were applied for qualitative variables. Odds ratios (OR) with 95% confidence intervals (CI) were calculated. Correlation was assessed using the Spearman rank coefficients (R). Receiver operating characteristic (ROC) curves were drawn and the areas under the ROC curves (AUROC) were calculated to assess the predictive value of investigated continuous variables. Logistic regression was applied to verify findings from bivariate comparisons. Logistic ORs with their 95% CIs were calculated. All tests were two-tailed. *p* value was set at 0.05.

## 3. Results

A study group comprised 30 men and 31 women at a median age of 62 (IQR 40–72) years; 57 (93%) of them required catecholamines on ICU admission and they had a median concentration of lactate of 3.2 (IQR 2.2–5.3) mmol/L. The remaining 4 (7%) patients required catecholamine infusion during the first day post-admission. Ten of the 61 individuals had lactate level <2 mmol/L initially but it increased within 24 h post-admission to satisfy the definition of shock. All subjects were mechanically ventilated on admission. A control group consisted of 36 men and 41 women at a median age of 63 (47–71) years; 64% of them received catecholamines and all of them were mechanically ventilated on ICU admission. The study group statistically significantly differed from the control group in terms of CRP and PCT levels, as well as APACHE II, SAPS II and SOFA scores (*p* < 0.05 for all) (Table 1). Mortality was 66% in the study group and 32% in the control group (OR = 3.96; 95% CI 1.94–8.07; *p* < 0.01). Basic characteristics of participants are shown in Table 1.

Basic parameters of whole blood analysis are shown in Table 2. Study group patients had statistically significantly lower lymphocyte (*p* < 0.01) and monocyte (*p* = 0.01) counts. Although there was no difference for red blood cell count, subjects with septic shock had significantly higher red cell distribution width compared to non-septic shock patients.

The median values (IQR) for NLR, PLR, MLR and PLT/MPV are depicted in Table 3. Septic patients had significantly higher NLR (*p* < 0.01). The difference for PLR was of a borderline significance (*p* = 0.07).

Investigating coefficients of correlation between the indices and selected parameters, we found that in patients with septic shock there were statistically significant associations between: PLR and PCT (*R* = −0.284, *p* = 0.04), PLR and lactate (*R* = −0.294, *p* = 0.02), MLR and CRP (*R* = −0.379, *p* = 0.002), MLR and PCT (*R* = −0.441, *p* = 0.001), PLT/MPV and PCT (*R* = −0.310, *p* = 0.03), PLT/MPV and lactate (*R* = −0.443, *p* = 0.001), PLT/MPV and SAPS II score (*R* = −0.330, *p* = 0.01), and PLT/MPV and SOFA score (*R* = −0.315, *p* = 0.01). No significant correlations were found in the control group.

ROC analysis revealed that NLR predicted the occurrence of septic shock with AUROC = 0.655 (95% CI 0.580–0.743); *p* < 0.01 (Table 4). AUROC for PLR was of a borderline statistical significance. MLR and PLT/MPV were impractical in prognostication from statistical point of view. CRP and PCT had higher diagnostic accuracy than NLR, with AUROC = 0.865 (95% CI 0.796–0.916); *p* < 0.001 and AUROC = 0.877 (95% CI 0.785–0.939); *p* < 0.001, respectively. Lactate level was also a significant predictor of septic shock, with AUROC = 0.659 (95% CI 0.570–0.740); *p* = 0.001. The combination of CRP with NLR increased the diagnostic accuracy of septic shock prediction to AUROC = 0.879 (95% CI 0.812–0.929); *p* < 0.0001. The combination of PCT with NLR kept diagnostic accuracy at a level of AUROC = 0.887 (95% CI 0.797–0.947); *p* < 0.0001. The combination of lactate with NLR slightly increased accuracy to AUROC = 0.673 (95% CI 0.584–0.753); *p* = 0.003. In logistic regression, only CRP and PCT were significant predictors of septic shock occurrence (Table 5).

In the study group, NLR, PLR, MLR and PLT/MPV were comparable between survivors and non-survivors: 20.7 [IQR 11.1–34.2] vs. 19.6 [IQR 10.2–32.8] (*p* = 0.79) for NLR, 332.4 [IQR 202.0–530.5] vs. 294.7 [IQR 105.7–495.9] (*p* = 0.47) for PLR, 1.02 [IQR 0.57–1.65] vs. 0.71 [0.34–1.18] (*p* = 0.15) for MLR, and 21.6 [IQR 15.0–34.4] vs. 17.0 [IQR 8.2–32.1] (*p* = 0.21) for PLT/MPV, respectively. In the control group, the indices were also comparable between survivors and deceased: 12.3 [IQR 7.3–17.6] vs. 11.8 [IQR 6.6–20.3] (*p* = 0.87) for NLR, 244.7 [IQR 154.6–393.7] vs. 227.2 [IQR 87.9–306.4] (*p* = 0.13) for PLR, 0.86 [IQR 0.40–1.12] vs. 0.88 [0.66–1.23] (*p* = 0.62) for MLR, and 22.23 [IQR 16.6–29.8] vs. 18.0 [IQR 9.5–23.1] (*p* = 0.06) for PLT/MPV, respectively.

All haemogram-derived indices failed to predict ICU mortality in patients with septic shock (Table 6). APACHE II predicted mortality with AUROC = 0.677 (95% CI 0.535–0.781); *p* = 0.02. SAPS II predicted mortality with AUROC = 0.699 (95% CI 0.569–0.809); *p* = 0.005. SOFA failed to predict mortality with AUROC = 0.606 (95% CI 0.474–0.728); *p* = 0.18. In logistic regression, all parameters were unable to predict the outcome (Table 7).

## 4. Discussion

In this single-centre retrospective study, we sought to verify the clinical usefulness of haemogram-derived indices in critically ill patients with sepsis-related multi-organ failure. We found that although high NLR was accurate in identifying patients at risk for septic shock, it failed to predict early mortality in this specific clinical scenario. PLR, MLR and PLT/MPV were impractical, both in screening and in prognostication. Interestingly, we confirmed that NLR could be a good adjunct to CRP or PCT to predict septic shock occurrence.

Sepsis is characterized by a dysregulated host response to infection. The alterations in the haemostatic system relate to the total number (and function) of white and red blood cells, and platelets. The most common features are leucocytosis (or less frequently, leukocytopenia), anaemia (or in the early period, increase in the RDW) and thrombocytopenia. The key cell types of the innate immune system and the first cellular line of defence against infection are neutrophils. During infection and endotoxaemia, a large number of neutrophils are produced. Demargination and enhanced bone marrow recruitment is of great importance. Activated cells secrete several enzymes, including acid phosphatase, myeloperodixadase and elastase, which cause tissue destruction. Depressed function of neutrophils in sepsis may cause their failure to phagocytize and clear the invading pathogens. They undergo subsequent apoptosis, which is beneficial, in contrast to lymphocytes. A persistent increase in the number of neutrophils indicates a lasting response to active infection. Lymphocytes are involved in adaptive immune response. Their response to infection relates to the number of total circulating lymphocytes and the different T-cell subpopulations. Lymphocyte apoptosis is rapidly increased in sepsis and lymphopenia may be a sign of devastating response to harm due to infection. Monocytes are a component of the host response that act as a link to the adaptive system via antigen presentation to lymphocytes. Platelet count is negatively correlated with MPV. Platelets with higher MPV have more secretory granules and larger surface area and therefore are more susceptible to activation.

NLR is a promising marker of inflammation and dysregulation in homeostasis. In our study, between-group difference in NLR was related to a significant decrease in lymphocyte count, whereas neutrophil count remained similar. High NLR, with a cut-off of 18.9, was a strong predictor of septic shock, with AUROC = 0.66. Noteworthy, this effect disappeared in multivariate analyses. Median NLRs were comparable between deceased patients and survivors in both, i.e., the study and control groups. NLR also failed to predict mortality in multivariate analyses. Jiang et al., in their systematic review and meta-analysis, found that, across eight studies, the diagnostic value of the NLR for the diagnosis of bacteraemia was comparable with our results, with AUROC of 0.69 (95% 0.65–0.73) [8]. Worth emphasizing is the fact of significant heterogeneity of the included studies (*I*^2^ = 91.5%). Moreover, one ought to realize that six of eight studies concerned patients at emergency departments with suspected community-based infection. In the ‘Discussion’, they underlined the need for using multiple combinations of biomarkers to enhance the clinical diagnosis of bacteraemia, including CRP, PCT and lactate. This conclusion is based on previous research [11,14,15]. In another meta-analysis, based on data from six studies, Russell and colleagues found that AUROC for the prediction of bacteraemia by NLR was slightly higher than in our investigations and reached 0.72 (95% CI 0.69–0.74) [9]. The optimum cut-off was 12.65 (OR = 4.1), which was significantly lower compared to our results.

The above meta-analyses constitute a certain averaging of the observations regarding the relationship between the haemogram-derived parameters and the occurrence of sepsis or bacteraemia. Much more interesting observations can be found in the analysis of individual original papers published in recent years. Gurol et al. [16] sought to clarify the cut-off values for NLR according to PCT level in patients with suspected bacteraemia or sepsis. While the overall correlation between NLR and PCT was poor (*R* = 0.26; *p* < 0.01), in patients with PCT within the range of 0.5–2 ng/mL, 2–10 ng/mL and >10 ng/mL, the NLR were: 11.8 ± 14, 13.2 ± 6.4 and 16.9 ± 6.6, respectively. The diagnostic accuracy in outcome prediction was acceptable, with AUC = 0.751 (95% CI 0.713–0.786). Although their results are different from our data, is should be underlined that the mean NLR in the cited paper was 8.1 ± 9.8, which was much lower compared with our patients, even from the control group. In addition, there was no clear time-point of blood sampling and there were many exclusion criteria for participants, including leukopenia, neutropenia and thrombocytopenia. De Jager and colleagues [17] retrospectively evaluated the ability of NLR to predict bacteraemia in patients on admission to the emergency department. Their revealed that the AUC was 0.73 (95% CI 0.66–0.81), which was lower compared with our results. Interestingly, the mean NLR was 20.9 ± 13.3 in patients with bacteraemia and 13.2 ± 14.1 in patients without bacteraemia, which was comparable with our observations. Although the values for CRP were also similar, the prognostication using this parameter was much better in our cohort (AUC = 0.62; 95% CI 0.54–0.70 vs. 0.86; 95% CI 0.8–0.92). NLR and PLR were significant predictors of infection in a comparable cohort of intensive care patients in Turkey [18]. However, the values of NLR, PLR as well as CRP and PCT in their study were completely incomparable with our data, which makes the comparison impractical. In other Turkish research [10], NLR was significantly higher in septic patients on ICU admission, compared to non-septic SIRS subjects (i.e., 11.5 vs. 10; *p* < 0.01), but in multivariate analysis it failed to predict sepsis (*p* = 0.14). Interestingly, the likelihood of sepsis was increased 18 times by the combination of CRP ≥4.0, lymphocyte count <0.45 and platelet count <150. In their study, NLR was comparable between survivors and deceased, which is in line with our data. A comprehensive investigation of the role of NLR and PLR in critically ill and injured patients was presented by Djordjevic et al. [19]. The mean APACHE II score was 22.3 ± 4 points and mortality reached 46%. The median NLR was 11 (IQR 7–15.7) and was higher in non-survivors (12.3; IQR 7.7–18) compared to survivors (9.9; IQR 6.2–13.8) (*p* = 0.001). NLR was higher in sub-groups of infectious subjects with higher mortality, i.e., 11.2 (IQR 7.9–17) in those with peritonitis (mortality 52.6%), 11.4 (IQR 6.5–15.7) in those with pancreatitis (mortality 58.2%). NLR was unrelated to the nature of infection and the results to blood cultures. PLR was unrelated to the underlying infectious condition and did not differ between survivors and non-survivors.

Literature data regarding MLR and PLT/MPV are scarce. Although both of them appear to be novel predictors of the compromised outcome, their role is more pronounced in systemic inflammation than in septic shock. Djordjevic et al. [19], for the first time, demonstrated that MPV/PLT was higher (*p* < 0.01) in non-survivors with sepsis and/or trauma hospitalized in the surgical ICU. In addition, MLR and MPV/PLT differed in terms of the nature of bacteraemia: patients with Gram-positive blood culture had significantly lower MPV/PLT compared to patients with Gram-negative and polymicrobial blood cultures. Individuals with Gram-negative, polymicrobial and negative blood cultures had significantly higher MLR values in comparison with those who had Gram-positive blood culture. Moreover, MLR significantly differed in terms of underlying infection and was the lowest in peritonitis (0.51) and trauma with sepsis (0.59), which was significantly lower compared with our data.

Full explanation of the above mentioned discrepancies is difficult. Of note, there are marked differences within the population under investigation regarding the underlying condition, clinical setting, severity of illness and many others. Our population seems to be quite specific compared to previous research in the field. The median scorings in APACHE II and SAPS II were 25 and 56 points in the study group, which were much higher than in the above cited papers. Multi-organ failure may significantly interfere with the observations regarding the relationship between NLR and mortality [20,21]. However, this drawback could be interpreted as the strength of our research. For the first time, we sought to investigate the clinical usefulness of NLR, PLR, MLR and MPV/PLT in subjects with high mortality suffering from septic shock. More important, however, are the observations that haemogram-derived indices significantly vary in the course of sepsis. These variations may result from the nature of sepsis, concomitant multi-organ failure or the applied treatment. In the study of Gharebaghi et al. [22], NLR increased in three consecutive days of the ICU stay, especially in the group of non-survivors (from 13.1 ± 1.7 to 17.2 ± 2.2). Hwang and colleagues [23] confirmed that both persistently low NLR (HR = 2.25) and persistently high NLR (HR = 2.65) were significant predictors of 28-day mortality in critically ill septic patients. In graphical presentation, the relationship between NLR and mortality was J-shaped. Finally, in the study of Riche et al. [24], the NLR at admission was significantly lower in patients who died before day 5 than in survivors (*p* = 0.01). In addition, from day 1 to 5, an increased NLR related to an increase in neutrophil count and a decrease in lymphocyte count was associated with late death (*p* = 0.003).

## 5. Study Limitations

One should bear in mind the potential limitations of our study. Firstly, this is a single-centre case-control study and may be biased with regard to the heterogeneous population and relatively small sample size. The results may be affected by the confounding effect of the data selection process. No power analysis was performed to assess the sample size. In addition, we did not take into consideration the origin of sepsis, the nature of bacteraemia and comorbid conditions. We lacked bacterial culture data, such as the percentage of positive culture per patient or the most common bacteria and their resistance profiles. This needs to be clarified in further research. Secondly, there may be bias with regard to the impact of multimodal personalized treatment, including antibiotics, source control techniques and supportive therapy. This may explain the lack of a link between investigated indices and the outcome, which was probably dependent on the applied treatment. Thirdly, as described above, there are significant variations in NLR during sepsis and we did not take into account this drawback.

## 6. Conclusions

Based on this negative study, we may assume that high NLR may be useful to identify patients with multi-organ failure due to septic shock, but this index should be interpreted along with CRP or procalcitonin levels to improve its diagnostic accuracy. NLR, PLR, MLR and PLT/MPV are not related with mortality in this specific clinical setting, even if assessed concomitantly with APACHE II, SAPS II or SOFA scorings.

## Figures and Tables

**Table 1:** Subjects’ basic characteristics.

Variable	Study Group	Control Group	*p*
Females	31 (51%)	41 (53%)	0.8
Age (years)	62 [40; 72]	63 [47; 71]	0.7
Lactate (mmol/L)	3.2 [2.2–5.3]	2.1 [1.5–3.6]	<0.01
C-Reactive Protein (mg/L)	160 [62–314]	19.3 [5.8–50.2]	<0.01
Procalcitonin (ng/mL)	3.32 [1.1–15.34]	0.19 [0.09–0.63]	<0.01
APACHE II (points)	25 [18–30]	16 [13–22]	<0.01
SAPS II (points)	56 [44–73]	30 [30–53]	<0.01
SOFA (points)	12 [9–14]	8 [7–11]	<0.01
Mortality	40 (66%)	25 (32%)	<0.01

Qualitative values are expressed as medians with their interquartile ranges [in brackets]; quantitative data are expressed as absolute values and percent (in brackets); APACHE II—Acute Physiology and Chronic Health Evaluation II; SAPS II—Simplified Acute Physiology Score II; SOFA—Sequential Organ Failure Assessment.

**Table 2:** Selected parameters of whole blood analysis.

Variable	Study Group	Control Group	*p*
White blood cell count (10^3^/µL)	15.0 [10.2–21.7]	12.8 [9.9–17.1]	0.19
Neutrophil count (10^3^/µL)	12.7 [9.1–10.4]	11.4 [8.2–15.6]	0.13
Lymphocyte count (10^3^/µL)	0.62 [0.39–1.04]	0.92 [0.67–1.42]	<0.01
Monocyte count (10^3^/µL)	0.61 [0.31–1.0]	0.88 [0.48–1.37]	0.01
Platelet count (10^3^/µL)	199 [110–294]	219 [163–290]	0.21
Mean platelet volume (fL)	10.9 [10.2–11.8]	10.6 [10.1–11.2]	0.06
Red blood cell count (10^6^/µL)	3.43 [2.97–4.17]	3.91 [ 3.24–4.36]	0.08
Red cell distribution width (%)	15.7 [14.3–17.5]	13.5 [12.9–15.0]	<0.01

Values are medians with their interquartile ranges [in brackets].

**Table 3:** Investigated haemogram-based indices.

Variable	Study Group	Control Group	*p*
NLR	20.5 [11.1–33.6]	12.9 [7.0–18.7]	<0.01
PLR	318.0 [135.1–525.8]	241.9 [147.4–356.2]	0.07
MLR	0.76 [0.36–1.41]	0.87 [0.44–1.24]	0.72
PLT/MPV	18.4 [12.2–30.4]	21.2 [15.1–29.5]	0.34

Values are medians with their interquartile ranges [in brackets]; PLR—platelet-to-lymphocyte ratio; NLR—neutrophil-to-lymphocyte ratio; MLR—monocyte-to-lymphocyte ratio; PLT/MPV—platelet count to mean platelet volume ratio.

**Table 4:** Septic shock prediction by investigated indices in bivariate analyses.

Variable	OR (95% CI); *p*	AUROC (95% CI); *p*	Cut-Off
NLR	1.04 (1.02–1.07); *p* = 0.001	0.655 (0.580–0.743); *p* < 0.01	>18.9
PLR	1.001 (1.00–1.003); *p* = 0.05	0.588 (0.501–0.671); *p* = 0.08	N/A
MLR	1.26 (0.87–1.81); *p* = 0.21	0.518 (0.431–0.604); *p* = 0.72	N/A
PLT/MCV	0.99 (0.97–1.03); *p* = 0.98	0.549 (0.460–0.635); *p* = 0.37	N/A

OR—odds ratio; CI—confidence interval; AUROC—area under the ROC curve; N/A—not applicable; PLR—platelet-to-lymphocyte ratio; NLR—neutrophil-to-lymphocyte ratio; MLR—monocyte-to-lymphocyte ratio; PLT/MPV—platelet count to mean platelet volume ratio.

**Table 5:** Septic shock prediction in multivariate analysis.

Variable	Logistic OR	95% Confidence Interval	*p*
Lactate (mmol/L)	0.84	0.61–1.16	0.30
C-Reactive Protein (mg/L)	1.01	1.01–1.02	0.03
Procalcitonin (ng/mL)	2.29	1.21–4.35	0.01
NLR	1.08	0.98–1.20	0.12
PLR	0.99	0.99–1.00	0.64
MLR	0.78	0.29–2.09	0.62
PLT/MPV	1.02	0.94–1.11	0.58
AUROC = 0.921 (95% CI 0.830–0.972); *p* < 0.0001			

OR—odds ratio; CI—confidence interval; AUROC—area under the ROC curve; PLR—platelet-to-lymphocyte ratio; NLR—neutrophil-to-lymphocyte ratio; MLR—monocyte-to-lymphocyte ratio; PLT/MPV—platelet count to mean platelet volume ratio.

**Table 6:** Mortality prediction in patients with septic shock by investigated indices in bivariate analyses.

Variable	OR (95% CI); *p*	AUROC (95% CI); *p*	Cut-Off
NLR	1.00 (0.97–1.03); *p* = 0.93	0.520 (0.390–0.649); *p* = 0.79	N/A
PLR	0.99 (0.99–1.00); *p* = 0.49	0.566 (0.425–0.683); *p* = 0.46	N/A
MLR	0.96 (0.62–1.47); *p* = 0.84	0.612 (0.480–0.733); *p* = 0.13	N/A
PLT/MCV	0.98 (0.94–1.01); *p* = 0.19	0.601 (0.463–0.729); *p* = 0.20	N/A

OR—odds ratio; CI—confidence interval; AUROC—area under the ROC curve; N/A—not applicable; PLR—platelet-to-lymphocyte ratio; NLR—neutrophil-to-lymphocyte ratio; MLR—monocyte-to-lymphocyte ratio; PLT/MPV—platelet count to mean platelet volume ratio.

**Table 7:** Mortality prediction in patients with septic shock in multivariate analysis.

Variable	Logistic OR	95% Confidence Interval	*p*
APACHE II (points)	1.06	0.94–1.19	0.33
SAPS II (points)	1.04	0.99–1.10	0.08
SOFA (points)	0.85	0.61–1.18	0.32
NLR	1.00	0.96–1.06	0.73
PLR	0.99	0.99–1.00	0.80
MLR	0.91	0.46–1.80	0.79
PLT/MPV	0.98	0.93–1.04	0.51
AUROC = 0.735 (95%CI 0.602–0.843); *p* < 0.01			

OR—odds ratio; CI—confidence interval; AUROC—area under the ROC curve; APACHE II—Acute Physiology and Chronic Health Evaluation II score; SAPS II—Simplified Acute Physiology Score II; SOFA—Sequential Organ Failure Assessment score; PLR—platelet-to-lymphocyte ratio; NLR—neutrophil-to-lymphocyte ratio; MLR—monocyte-to-lymphocyte ratio; PLT/MPV—platelet count to mean platelet volume ratio.
